# The role of dental public health in patient, public and the professions' safety: insights and case studies from England

**DOI:** 10.1038/s41415-026-0189-3

**Published:** 2026-11-13

**Authors:** Sarah Kaddour, Lwazi Sibanda, Sally Eapen Simon, Samantha Watt, Rakhee Patel

**Affiliations:** 482568156927670659353 NHS England, West Midlands, United Kingdom; 064681047798709998317 Primary Care and Public Health Commissioning Directorate, NHS England, London, United Kingdom; 965685826310463906583 Public Health Directorate, NHS England, North East and Yorkshire, United Kingdom; 910787109984288322738 Faculty of Dentistry, Oral and Craniofacial Sciences, King´s College London, London, United Kingdom

## Abstract

**Introduction** Dental public health (DPH) is a clinical dental specialty that works across six domains and the three pillars of public health, including health protection. Across the UK, the DPH speciality workforce plays a vital role in the management of dental health protection cases, utilising their system-level knowledge and clinical leadership to address complex health protection incidents in dental setting. This paper is one of two papers dealing with health protection as part of this Themed Issue on the speciality and aims to provide insights to the systems leadership role it plays within health protection across the UK. The second paper is focussed on antimicrobial resistance, which is another key element of protecting the health of our population.

**Methods** This paper provides a narrative overview of the role of dental public health and includes case studies focussed on the contribution of specialist leadership during the COVID-19 pandemic response and the management of a dental case involving a practice with infection prevention control concerns.

**Case studies** Through these case studies, the proactive and wide impact the speciality has in the protection of patients, the public and the dental workforce is highlighted.

**Conclusions** The management of complex dental incidents, with health protection concerns requires robust, timely, and evidence-informed system response. Specialists in DPH provide critical clinical and system leadership, enabling partnership working and influencing coordinated actions across organisational boundaries. Their specialist understanding of the unique combination of dentistry and public health is crucial for patients, public, and healthcare workforce protection.

## Key points

- The whole oral health team has a key role in multiple aspects of health protection and outbreak management. Their role includes adherence to robust infection prevention control measures, local protocols to plan for outbreak scenarios and undertaking risk assessments.
- When there is a breach of standards, and an incident or a risk of one occurs, dental public health teams work with local health protection and dental systems to develop a proportionate response to the level of risk and ensure patients, the public and professionals are safe.
- Dental public health is key in pandemic response and the planning of emergency preparedness.

## Introduction

Dental public health (DPH) is the clinical dental speciality concerned with prevention of oral disease, promoting oral health and improving quality of life, by working across the three main pillars of public health: health improvement, healthcare public health and health protection.^1^ The speciality spans across three further domains that form its core work; health intelligence, workforce development and academia.^2^ This paper is one of two health protection papers in this Themed Issue. This paper highlights the role of the DPH workforce across health protection, with antimicrobial resistance as the focus of a further paper within the issue.

Health protection is ‘the protection of individuals, groups and populations through the effective collaboration of experts in identifying, preventing and mitigating the impacts of infectious diseases and of environmental, chemical and radiological threats.^3^ Therefore, health protection covers a wide range of areas, including communicable disease control, immunisation, screening and emergency prevention preparedness and response.

The speciality focuses on protecting and improving oral health at a population level. To enable this, epidemiological data and intelligence, surveillance and evidence-based preventative strategies are used to address oral health diseases and oral health inequalities. Part of the fundamental remit of the speciality is the contribution to the control of infectious disease, emergency preparedness, and responses to environmental hazards, all in relation to dental settings and the dental workforce.^4^^,^^5^

Dental care providers and oral health teams have a duty of care to their patients and team. This is met by ensuring that appropriate governance arrangements for risk management and national infection prevention control standards are met.^6^ This duty is part of the professional regulatory position, and employee's healthcare organisation.^5^ Furthermore, this duty is aligned to relevant legal frameworks, including responsibilities for safety that employers have.

However, breaches of health protection standards and safety incidents do occur. In terms of health protection, and risk, it is considered that dental professionals undertake high volume and at times high risk, exposure prone procedures, in as much as the dental setting can be an environment where the spread of communicable and non-communicable disease may occur. Communicable diseases (e.g., hepatitis B, hepatitis C, human immunodeficiency virus) and respiratory pathogens (e.g., tuberculosis, COVID-19) can spread via bilateral risks (from patients to staff and staff to patients). Sources can also occur from the practice to patients/staff via the structural sources such as waterlines (e.g., *Legionella*) and hazardous materials and waste management procedures (e.g. amalgam waste). There are factors that increase the health protection risks in dentistry including clinician behaviour (e.g., breakdown of infection prevention control procedures), the dental environment, (e.g., close working environments, aerosol generating procedures, surgical procedures) and environmental hazards (e.g., the management of waste).

As patient and workforce safety are inherently linked, when there is a failure to meet standards in a dental setting, due to the high-risk nature of the environment, this poses a risk of the transmission of disease into the wider community, to patient‘s households, workplaces and other community settings.^7^

The majority of incidents within or linked to dental settings that occur can be contained and managed; however, there have been high profile incidents covered in the media that did result in harm. The most prominent being the ‘D'Mello case' which became one of the largest patient recall exercises in National Health Service (NHS) history.

In this instance, timely, appropriate, and evidence-informed system responses with appropriate clinical expertise is vital. Dental health protection cases are particularly unique compared to wider healthcare because of the nature of dental care being surgery in a primary care setting and in domiciliary care. Hence, having clinical dental expertise in the form of dental advisors and dental public health to explain the function of dentistry, understand the nuances of the care provided and ensuring the inclusion of key stakeholders in managing cases is critical. This ensures that the response is immediate and actions proportionate to the level of risk.

**Fig. 1:**
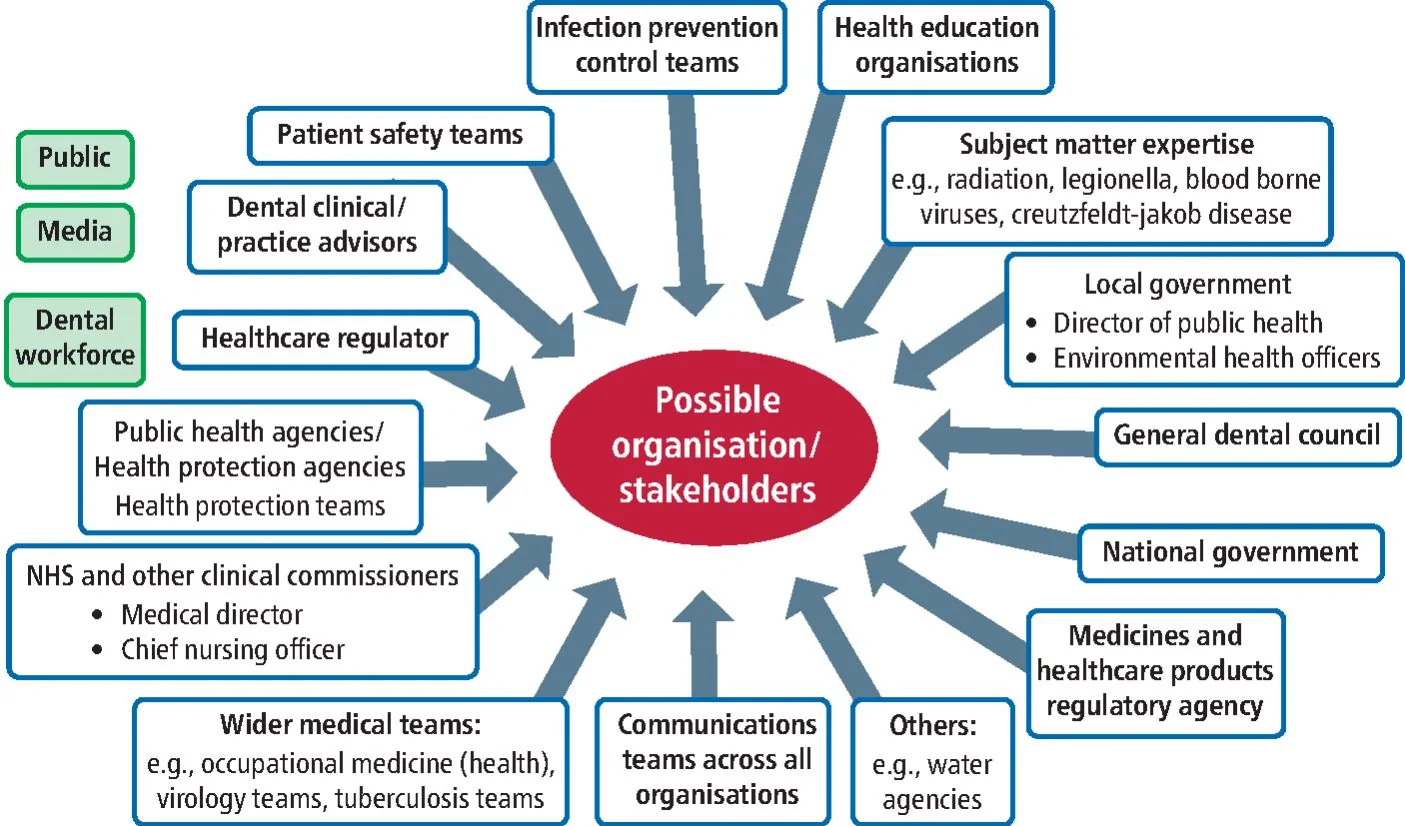
Non-exhaustive list of possible stakeholders and some of the key national organisations/stakeholders in the UK that DPH engage with during the management of dental health protection

There are differences in the stakeholders, policy and legislature across the UK, and therefore differences in responses to health protection cases. Key organisations and their roles across the UK are shown in Table 1 for the four nations. There are a range of stakeholders across the UK that may need to be involved in the management of dental health protection cases, as shown in Figure 1; this is not an exhaustive list, and their involvement will depend on the nature of the incident, and due to reorganisations, stakeholders and organisational roles and responsibilities may change. However, the role of dental public health in each nation is largely the same- to offer expert public health advice and act as a critical friend to management of the case.

**Table 1:** Summary of proactive (preventative) and responsive (reactive) management for dental health protection cases

**Proactive (preventative)**	**Responsive (reactive)**
Infection prevention control protocols Training and Continuing Professional Development (CPD) Occupational health checks to obtain Exposure Prone Procedure (EPP) Clearance Dental inspections Tabletop exercise (team planning exercises)	Incident and outbreak management Patient notification (lookback) exercises

Given the system leadership nature of DPH, DPH specialists work as part of an incident management team (IMT). The primary aim of an IMT is ‘to protect public health by identifying the source and cause of infection and transmission, and deciding upon, and coordinating, control measures to reduce transmission and outbreak-related harm'. IMTs are multidisciplinary and multiagency groups, usually chaired by a consultant in health protection, with the consultant in DPH providing critical dental leadership. Consultants in DPH will consult and present to the IMT-relevant dental evidence-base and policy context as described in Figure 1, for the situations within the IMT.

It should be noted that system and organisational change add complexity to the established incident management processes and relationships. It is imperative that the DPH workforce are supported to maintain their expert systems leadership roles to engage and co-ordinate stakeholders involved in incident management. The speciality provides strategic leadership, clinical support and advice to better inform and enable a system response to incidents, cases and outbreak management linked to dental settings, the aim of which is to protect patient, public and the healthcare workforce. This is done in collaboration with multiple agencies and partners across the healthcare system, local government (local authorities) and dental stakeholders. The loss of DPH expertise through repeated reorganisations poses a significant risk to the healthcare and public health system responsiveness to dental health protection cases and therefore presents a significant risk to patient and public safety.

To further conceptualise the role of DPH in health protection and how cases are managed, two cases are presented in this paper. The first explored a national incident response, highlighting an example of emergency preparedness and response, whilst the second related to the management of a local patient safety incident. Both cases highlight the role and importance DPH has at both a local and wider system level.

## Case study 1: the London dental approach in managing the COVID-19 pandemic

The COVID-19 pandemic had a profound impact on dentistry. On 25 March 2020, during the first COVID-19 wave, the UK government suspended all non-urgent NHS dental treatment including orthodontic treatment, aerosol-generating procedures and face-to-face urgent dental care. Providers were required to establish remote urgent care services via telephone triage, whilst urgent dental care systems were setup for face-to-face urgent dental care, of which there were over 600 in England.^8^^,^^9^ In recovery from the ‘first wave', on 28 May 2020, the Office of the Chief Dental Officer England (OCDO England) and NHS England (NHSE) asked all dental practices to resume a transition to face-to-face service provision from 8 June 2020.^10^ A risk-based approach meant services opened with extensive changes to infection prevention contro (IPC) procedures, resulting in significant reduction in patient throughput.

Throughout the pandemic, guidance was frequently evolving from UK Government, Public Health England (PHE), NHSE and OCDO England. A standard operating procedure was released by OCDO England to support safe remobilisation of the dental workforce, later renamed ‘transition to recovery'.^11^ PHE published guidance on infection prevention and control for COVID-19 in January 2020, guidance that iteratively updated as the pandemic progressed. In October 2020, a dental appendix was published to support the remobilisation and maintenance of this service across the UK*.*^12^ This was in addition to regularly revised national COVID-19 stay at home guidance, health professional guidance amid others.^13^

In England, regional coronavirus response cells (CRCs) were developed. These were led by health protection teams and had overall responsibility in regions in the approach of the acute response including case and outbreak management, enquiries, contact tracing and communicating with stakeholders. DPH teams provided strategic and clinical support to the national and regional CRCs and worked collaboratively with NHSE IPC teams to develop inspection protocols that were proportionate to the COVID-19 risk in dental settings.

During the ‘second wave' in the last calendar quarter of 2020, there was a rising tide of community-level infection across the UK. This resulted in significant increases in the number of cases, situations and enquiries that were being received by regional CRCs across all settings. Amidst a surge in cases and high levels of viral community transmission,^12^ coupled with reductions in dental access, capability and capacity, increasing anxiety and confusion in navigating a myriad of new policy and guidance was becoming apparent amongst dental professionals.^14^^,^^15^

**Fig. 2:**
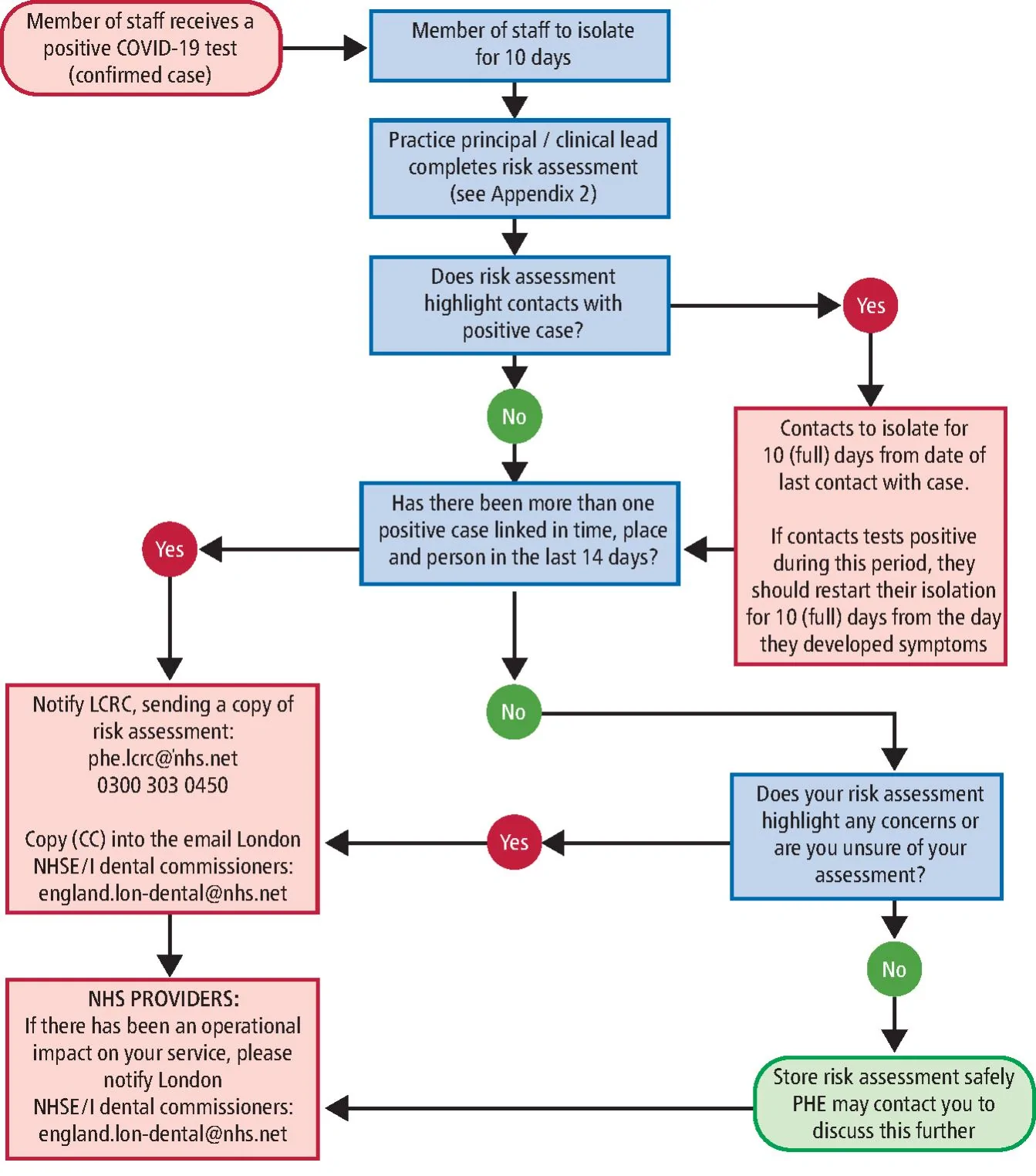
Extract from the dental COVID-19 toolkit

Auditing by DPH of confirmed cases that were linked in place, time or person within dental practices were found to be mainly due to staff-to-staff transmission. Enquiries relating to suspected and confirmed cases of COVID-19 linked to dental practices highlighted confusion in case management and prevention of outbreaks in part due to conflicting guidance documents. In response, the DPH team in London with stakeholders agreed that a proactive solution was required to collate and interpret the rapidly changing national guidance and policy into a locally relevant, user-friendly and succinct document (Fig. 2). The toolkit would in turn be communicated across by a range of stakeholders across different organisations. This was an opportunity to develop a real-world solution to expediting the translation of policies and guidance across the profession and to demonstrate an example of responsive DPH in action.

The toolkit and associated resources were disseminated to dental teams via local dental committees, Health Education England (HEE as it was known at the time), PHE and NHS England networks. Documents were also shared with other dental public health teams, regionally and nationally, which they amended according to respective arrangements and implemented locally. Supported by HEE, a webinar was hosted for dental teams across London to discuss use and application of the toolkit. This webinar, which received positive feedback, also highlighted the specific roles of the dental public health team, PHE and NHSE in outbreak management and provided an open forum in which dental teams could clarify concerns and ask questions.

The ongoing work by the dental public health team to support dental teams during the COVID-19 response increased awareness of the speciality and created opportunities to share learning. Throughout the response, multi-sector working was adopted and opportunities for collaboration were created.

## Case study 2: ensuring patient safety approaches at a period of change in the Northeast and Yorkshire region

Periods of reorganisation and loss of staff pose significant risk to health protection. In England this has been particularly challenging in the last few years. In October 2021, PHE was disbanded, and health protection function was moved to the newly formed UK Health Security Agency. Unlike other parts of public health, dental public health was moved from PHE to NHSE regions. In April 2023, commissioning responsibilities began to be delegated from NHSE regional commissioning teams to emerging local integrated care boards (ICBs). This was a phased transfer, which included dental services and infection prevention control. In relation to incident management, the turbulence of transfer of commissioning responsibilities and the absence of dental team specific guidance and processes for reporting of incidents presented a reporting risk. This was further compounded by a period of transition in NHS policy on the management of patient safety incidents from the Serious Incident Framework to the Patient Safety Incident Response Framework (PSIRF).^16^ Organisational roles, governance and reporting processes became unclear.

In 2024, a serious incident occurred in primary dental care which warranted an immediate response. With system-level knowledge, DPH specialist leadership was pivotal in highlighting the risk and the consequent facilitation of immediate management of the incident:

1. Collaborative working between the quality lead, dental clinical leadership and UK Health Security Agency resulted in the development of a risk assessment framework, patient communication and acknowledgement of the importance of protecting the health and wellbeing of staff involved. It was important patient communication reflected the NHS legal obligation in relation to the duty of candour describing the risk of potential harm
2. Collaborative working with the ICB patient safety speciality, dental clinical leadership and dental commissioning colleagues from the ICB led to development of an interim reporting mechanism. Immediate learning from this incident resulted in agreed reporting protocols which included key contact details and defined organisational roles and responsibilities regarding incident management in primary dental care. This has secured timely and appropriate management of incidents.

There was a national and regional expectation for dental providers to work to the NHS PSIRF guidance. However, there was no specific dental guidance that existed to help dental teams understand their roles and responsibilities concerning reporting and managing incidents. In March 2025 the OCDO published a bulletin describing patient safety including key documentation to support dental practices in reporting, managing and learning from patient safety incidents.^14^ While interim reporting mechanisms were in place for dental practices locally, there were concerns that dental colleagues were not aware of the updates to patient safety policy including the launch of the PSIRF. This bulletin was instrumental in providing clarification from a national perspective on patient safety in dental practices and provided an excellent opportunity to develop local information for dental practices on patient safety events.

DPH subsequently worked with the ICB patient safety speciality to develop bespoke guidance for dental teams to support reporting and management of patient safety events including a dental team briefing, summary page and guidance on the reporting platform ‘Learning From Patient Safety Events'.^17^ In addition, information on patient safety events for service specifications were revised to ensure compliance of newly commissioned services to national and local guidance. DPH leadership has been instrumental in the immediate management of the incident, but more importantly in the development of regional guidance which is an example of best practice.

## Discussion

There are many types of incidents that may occur, or potentially occur in dental settings, which can be considered as ‘proactive' or ‘reactive', as highlighted in Table 1. It is imperative that robust standards, policies and procedures are in place to prevent, mitigate or manage risk. This risk may be a breach of infection, prevention and control procedures or it could be from organisational change resulting in a period where clear roles and responsibilities around incident management in the dental setting aren't clarified.

Inevitably, incidents and accidents will occur. There are a multitude of stakeholders who need to work together, share wisdom and expertise to protect individuals and consequences from potential consequences of such events, as highlighted in Figure 1.

This will prove more challenging during organisation and system change. Dental public health can act as a ‘glue' through such changes, driving system cohesiveness and acting as hybrid system leaders with the aim of protecting patients, the public and dental professionals now and in the future.

## Conclusion

This paper presents examples that demonstrate the strategic contributions of the DPH speciality workforce to maintaining public, professional and patient safety.

The management of patient safety and health protection incidents in dental settings is inherently complex and requires a robust, timely, appropriate, and evidence-informed system response. Oral health teams have a key role in multiple aspects of health protection and outbreak management, including adherence to national antimicrobial prescribing guidelines and robust infection prevention control measures.

When an incident does occur, DPH specialist leadership plays a pivotal role in orchestrating such responses through partnership working, multidisciplinary collaboration, and effective system integration. By understanding both the clinical and public health dimensions and the intersections within the wider health system, DPH specialists can influence, steer, and align actions that protect patients, the public, and the healthcare workforce.
